# Shield effect of resistance jumping training on biomechanical parameters in gastrocnemius muscles of diabetic animals

**DOI:** 10.1186/1758-5996-7-S1-A12

**Published:** 2015-11-11

**Authors:** Deniele Bezerra Lós, Marcos Paulo Galdino Coutinho, Cybelle da Silva Nery, Camilla Rodrigues de Souza Silva, Suzy Kelly Ferreira Silvestre da Silva, Rafael Dornelas e Silva, Elvis da Silva, Rodrigues de Freitas, Waydja Lânia Virgínia de Araújo Marinho, Ana Cristina Falcão Esteves

**Affiliations:** 1 Universidade Federal de Pernambuco, Recife, Brazil

## Background

Chronic hyperglycemia resulting from diabetes mellitus undertakes several body cells through the formation of advanced glycation end products and activation of the polyol pathway (Brownlle, 2005), promoting reduction of electrical activity in skeletal muscle and the regenerative capacity of its cells, as well as impaired contractility and increased tissue stiffness. In turn, the amplify muscle work during exercise is able to elicit several essential biochemical reactions for hypertrophy, power gain and enhance muscle function (Andersen et al., 1996, 1997, 2004, 2005).

## Objective

To evaluate the biomechanical changes of muscles affected by diabetic state and the effect of resistance training on the biomechanical parameters.

## Materials and methods

65 male Wistar rats were divided into four groups: Sedentary Control (GCS, n=11), Trained Control (GCT, n=14), Sedentary Diabetic (GDS, n=19) and Trained Diabetic (GDT, n=21). GDS and GDT animals were administered with an intraperitoneal dose of streptozotocin to induce diabetes, which was confirmed by measurement of blood glucose fasting on the 3rd and 7th day post induction. GCT and GDT animals were submitted to an overload jumping exercise program of up to 50% body weight, 5 times a week during 9 weeks. At the end of the exercise period, the animals of all groups were anesthetized to collect the gastrocnemius muscles, keeping preserved its proximal and distal insertion into the femur and the distal insertion on the calcaneus for the tensile test of these muscles. Statistical analysis was performed by analysis of variance (ANOVA), using a significance level of 5%.

## Results

The mechanical test of the gastrocnemius-plant complex demonstrated in diabetic animals (GDS) lower values in biomechanical parameters: maximum strength (26.5±9.74 vs 51.5±9.21, p <0.05) deformation (10.2±3.30 vs 17.85±5.75, p <0.05), energy/area (4.90±2.21 vs 7.57±2.59, p <0.05 ), stiffness (1.05±0.36 vs 2.05±0.36, p <0.05) and cross-sectional area (30.13±7.48 vs 67.49±16.37, p < 0.05) compared to GCS, with an increase in maximum strength parameters (37.71±3.63 vs 26.5±9.74, p <0.05) and stiffness (1.5±0.14 vs 1 05±0.36, p <0.05) compared to the GDT.

## Conclusion

It has been concluded that the resistance jump training has improved the values of maximum strength and stiffness parameters of the gastrocnemius complex plant in diabetic animals.

